# Design of Dopant and Lead-Free Novel Perovskite Solar Cell for 16.85% Efficiency

**DOI:** 10.3390/polym13132110

**Published:** 2021-06-27

**Authors:** Syed Abdul Moiz, Ahmed N. M. Alahmadi

**Affiliations:** Department of Electrical Engineering, Umm Al Qura University, Makkah 21955, Saudi Arabia; anmahmadi@uqu.edu.sa

**Keywords:** solar cell, photovoltaic response, perovskite, lead-free, dopant-free, Cs_2_TiBr_6_, NPB, PCBM

## Abstract

Halide based perovskite offers numerous advantages such as high-efficiency, low-cost, and simple fabrication for flexible solar cells. However, long-term stability as well as environmentally green lead-free applications are the real challenges for their commercialization. Generally, the best reported perovskite solar cells are composed of toxic lead (Pb) and unstable polymer as the absorber and electron/hole-transport layer, respectively. Therefore, in this study, we proposed and simulated the photovoltaic responses of lead-free absorber such as cesium titanium (IV) bromide, Cs_2_TiBr_6_ with dopant free electron phenyl-C_61_-butyric acid methyl ester (PCBM), and dopant free hole transport layer N,N′-Di(1-naphthyl)-N,N′-diphenyl-(1,1′-biphenyl)-4,4′-diamine (NPB) for the Ag/BCP/PCBM/Cs_2_TiBr_6_/NPB/ITO based perovskite solar cell. After comprehensive optimization of each layer through vigorous simulations with the help of software SCAPS 1D, it is observed that the proposed solar cell can yield maximum power-conversion efficiency up to 16.85%. This efficiency is slightly better than the previously reported power-conversion efficiency of a similar type of perovskite solar cell. We believe that the outcome of this study will not only improve our knowledge, but also triggers further investigation for the dopant and lead-free perovskite solar cell.

## 1. Introduction

From the last few years, the power-conversion efficiency of perovskite solar cell has jumped from 3.8 to 25.5%, showing a remarkable progress in the history of photovoltaic industry [1]. Among these perovskite materials, methylammonium lead iodide (MAPbI_3_) has shown an excellent performance for photovoltaic applications. Unfortunately, lead is a highly toxic material and its presence in the environment can seriously destroy our soil, water, and other natural resources [2]. Hence, it is highly recommended to replace lead with other lead-free perovskite materials for future photovoltaic applications.

Recently, a novel lead-free perovskite compound such as cesium titanium (IV) bromide (Cs_2_TiBr_6_) is being very popular and many researchers believe that this perovskite material has the full potential to replace MAPbI_3_ for photovoltaic applications [3,4,5]. The Cs_2_TiBr_6_ perovskite offers excellent electrical, optical, and photovoltaic responses and similarly the incorporation of stable titanium (Ti) formulate Cs_2_TiBr_6_ a robust perovskite to tolerate higher thermal, electrical, environmental, and irradiation stresses [6].

For an efficient perovskite solar cell, the proper selection of both electron and hole-transport material is also very crucial. In this study, PCBM is used as a dopant free electron-transport layer with absorber Cs_2_TiBr_6_ for the novel perovskite solar cell. Generally, PCBM based fullerenes and their derivatives demonstrate excellent electron transport parameters which cause the reduction of the hysteresis of the photovoltaic responses [7,8]. For most cases, dopant-free bathocuproine (BCP) as a buffer layer between the cathode and PCBM is highly recommended to further improve the hysteresis, power-conversion efficiency, and stability of the perovskite solar cell [9]. Therefore, both BCP/PCBM combinations were used as an electron-transport layer for the proposed perovskite solar cell.

For the hole-transport layer, NPB (NPB or NPD both these abbreviations are frequently used for the same material) is a highly stable and commonly reported material for the perovskite solar cell [10,11]. The improved stability response of NPB compared to the other organic/polymer hole transport layer is mainly due to the hydrophobic nature of the NPB layer [12,13]. However, the doping of NPB may initiate many complex chemical processes leading to long-term stability issues for photovoltaic applications especially at a higher temperature [14,15,16,17]. Therefore, the dopant free NPB as a hole-transport layer was used for the current proposed perovskite solar cell.

**Figure 1:**
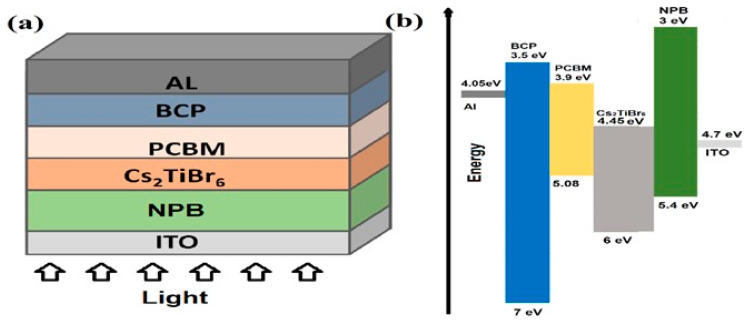
(**a**) The schematic structure, and (**b**) the energy-band alignment diagram of the proposed Ag/BCP/PCBM/Cs_2_TiBr_6_/NPB/ITO solar cell.

Figure 1a,b depicts the schematic structure and the energy-band alignment diagram for the proposed p-i-n type perovskite solar cell, where ohmic Ag and ITO contacts are used as front-end cathode and back-end anode, respectively. The band alignment, as shown in Figure 1b, is nearly consistent with the natural flow of photocarrier transport in both the conduction as well as the valence band, which is the first step towards the design of an efficient solar cell for maximum photovoltaic response.

Simulation and modelling are one of the simplest, low-cost, and highly regarded methods for the comprehensive investigation and optimization for photovoltaic response [18]. In the literature, various simulation and modelling tools are available and here the solar cell capacitance simulator-1 dimension (SCAPS 1D) is selected for this study. The SCAPS 1D is a general-purpose tool and highly recommended by various research groups for the simulation and modelling of perovskite solar cell [19,20]. Therefore, in this study, we proposed, designed, simulated, and optimized the novel lead-free and dopant-free perovskite solar cell as Ag/BCP/PCBM/Cs_2_TiBr_6_/NPB/ITO for maximum power-conversion efficiency with the help of the SCAPS 1D software.

## 2. Simulation Method and Device Parameters

The comprehensive simulation of the proposed lead and dopant free solar cell is performed using SCAPS 1D (version 3.3.07), as discussed above. SCAPS 1D is an excellent simulation platform, which is used to simulate the various types of one-dimension photovoltaic response. Broadly speaking, SCAPS 1D implements a set of photovoltaic models to simulate any type of photovoltaic response [19,20,21].

SCAPS 1D offers an interface to couple the fundamental photovoltaic equations governed by the user-defined geometry and material parameters for each layer. The fundamental equations used in these simulations are Poisson equations, device continuity equations, drift-diffusion charge transport model, recombination losses with defects model, optical absorption models, etc. These fundamental models can be further explored as:

1. Poisson model states that the one-dimension (*x*) Laplacian of the electrostatic potential field (φ) is equal to the ratio of total volume charge density and the permittivity
  (1) $$\frac{d^{2}\emptyset \left(x\right)}{dx^{2}}=\frac{q}{\in_{o}\in_{r}}\;\left(p\left(x\right)-n\left(x\right)+N_{D}-N_{A}+\rho_{p}-\rho_{n}\right)$$
  where *q* is the electronic charge (1.602 × 10^−19^ C), ε_0_ is the permittivity of vacuumed, ε*_r_* is the relative semiconductor permittivity, *N_D_*/*N_A_* are the shallow donor/acceptor impurity density, *n*(*x*)/*p*(*x*) are the electron/hole density at a position *x*, and *ρ_n_*/*ρ_p_* are the electron/hole density distribution.
2. The device continuity model states that change in the electron/hole current density (*J_n_*/*J_p_*) over a specific time as a function of position is equal to the result of generation (*G*) and the recombination (*R*) of electron/hole, respectively.
  (2) $$\frac{dJ_{n}}{dx}=G-R$$
  (3) $$\frac{dJ_{p}}{dx}=G-R$$
3. The semiconductor charge transport model describes that the total electron/hole current density (*J*) is the sum of electron/hole drift and diffusion current density
  (4) $$J=J_{n}+J_{p}$$
  (5) $$J_{n}=D_{n}\;\frac{dn}{dx}+\mu_{n\;}n\frac{d\emptyset }{dx}$$
  (6) $$J_{p}=D_{p}\;\frac{dp}{dx}+\mu_{p\;}p\frac{d\emptyset }{dx}$$
  where *D_n_*/*D_p_* are the electron/hole diffusion coefficient and *µ_n_*/*µ_p_* are the electron/hole mobility, respectively.
4. For the optical absorption coefficient, SCAPS offers different options for the calculation of the absorption coefficient α (λ), but in this study, we use the following equation depending on the relation of photons (*h* is the plank constant and *ν* is the photon frequency) and perovskite (as a absorber layer) energy bandgap (*E_g_*)
  (7) $$\alpha \;(\lambda )=\left(A+\frac{B}{h\nu }\right)\;\sqrt{h\nu -E_{g}}$$

Further detailed information about the simulation methodology can be found in our previous published paper [22]. All the materials parameters are extracted from the reported literature for BCP, PCBM, Cs_2_TiBr_6_, and NPB, which are listed in Table 1 [22,23,24,25,26,27,28]. Similarly, for photovoltaic characterization, the proposed device was simulated under solar illumination of air mass AM 1.5 G at 1 sun photons intensity (1000 W/m^2^), where ambient temperature of 300 K was used for photovoltaic simulation.

## 3. Results and Discussion

As we have selected undoped materials, therefore the thickness of each layer was only optimized for the current proposed perovskite solar cell. The film thickness of hole-transport layer, perovskite absorber, and electron-transport layer play a very crucial role to optimize the overall photovoltaic response of perovskite solar cell. Both the electron-transport layer and hole-transport layer interact not only with the perovskite layer but also with their respective electrode materials. The interaction between PCBM and cathode interface is improved by incorporating a very thin layer of BCP with already reported optimized parameters as shown in Table 1, therefore the BCP layer is not further optimized in this study.

### 3.1. Thickness Optimization of Electron Transport Layer

**Figure 2:**
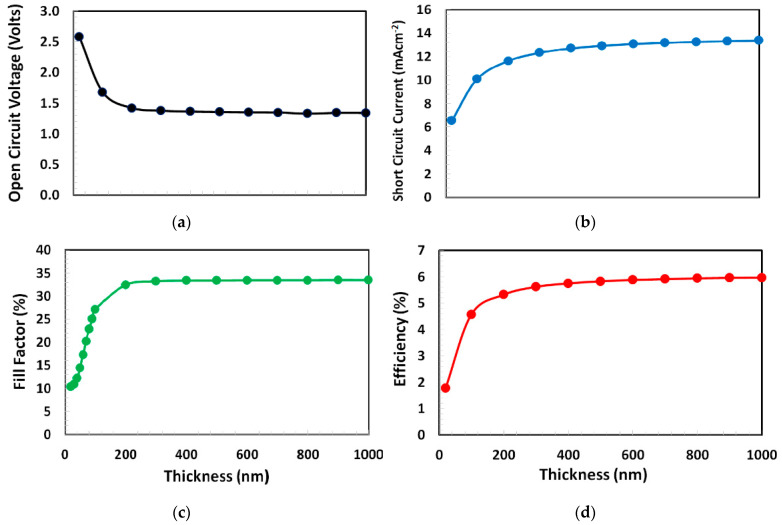
(**a**) Open-circuit voltage, (**b**) short-circuit current, (**c**) fill-factor, and (**d**) power-conversion efficiency of proposed solar cell Ag/BCP/PCBM/Cs_2_TiBr_6_/NPB/ITO as a function of PCBM layer thickness from 10 to 1000 nm.

For the non-inverted perovskite solar cell, the PCBM film-thickness depends on many factors such as charge collection from the perovskite layer, perovskite surface traps density, electron/hole recombination, carrier transport process, and electrode surface roughness. Therefore, in the first stage, the thickness of PCBM (as an electron-transport layer) was optimized for the proposed solar cell. In order to determine the optimize PCBM thickness for the proposed solar cell, we performed a series of photo current-voltage simulations and calculated the photovoltaic parameters such as open-circuit voltage, short-circuit current, fill-factor, and power-conversion efficiency by varying the PCBM thickness from 10 to 1000 nm, as shown in Figure 2. From the figure, it is observed that the open-circuit voltage sharply decreases from 2.5 volts and then saturates at close to 1.5 volts. While the short-circuit current, fill-factor, and most importantly power-conversion efficiency follow different trends with respect to the open-circuit voltage, where these responses sharply rise and then saturate at nearly 400 nm of PCBM thickness, as shown in Figure 2. Thus, it can be inferred from the above trends that the 400 nm is the optimum thickness of PCBM as an electron-transport layer for the proposed solar cell.

Generally, it is believed that the thinner PCBM layer may lead towards an improved charge transport process for the solar cell [29]. On the other hand, the solar cell is inherently a diode in nature and its photovoltaic response also depends on diode parameters such as the diode ideality factor, barrier-height, threshold-voltage, series resistance, shunt resistance, reverse saturation current, etc. and these parameters become further complex for the space-charge limited behavior of polymer diode [30,31,32,33,34]. Our simulation result shows that the thicker PCBM improves the device performance, which can be justified due to the better diode properties as it is also reported by many other researchers for the PCBM layer [35,36].

### 3.2. Thickness Optimization of Hole Transport Layer

By incorporating the optimized thickness of PCBM (400 nm), the thickness of NPB was optimized using the same material parameters, as shown in Table 1. Similar to other conducting polymers, the variable-range hopping charge-transport process in NPB is also complicated in nature and depends on the film thickness of the polymer [37]. Similarly, the nature and density of traps states that perovskite-polymer interfaces are very crucial to define the transport through the polymer. Therefore, the interface trap states between the polymer and perovskite absorber layer are introduced in the simulation, where detailed information about the interface trap states are listed in Table 2.

**Figure 3:**
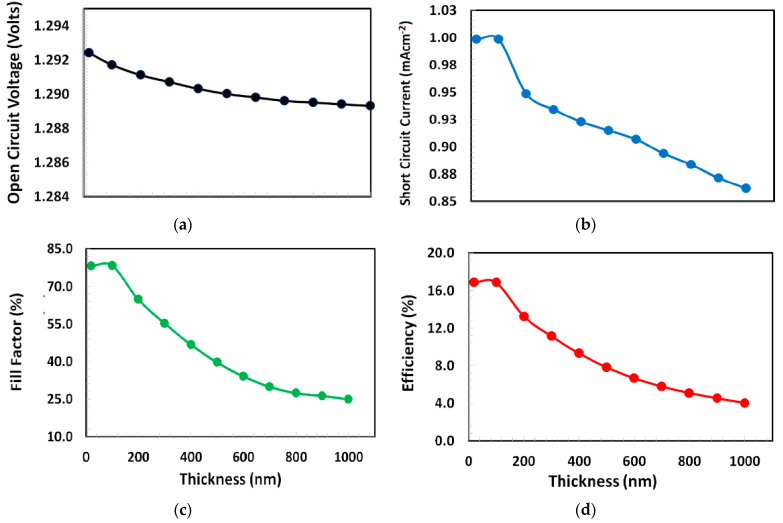
(**a**) Open-circuit voltage, (**b**) short-circuit current, (**c**) fill-factor, and (**d**) power-conversion efficiency of proposed solar cell Ag/BCP/PCBM/Cs_2_TiBr_6_/NPB/ITO as a function of NPB layer thickness varied from 10 to 1000 nm.

In order to optimize the thickness of NPB as the hole-transport layer a series of simulations were performed and the photovoltaic responses were determined by varying the thickness of NPB from 10 to 1000 nm and the obtained photovoltaic parameters such as open-circuit voltage, short-circuit current, fill-factor, and power-conversion efficiency are shown in Figure 3. The figures clearly demonstrate that the open-circuit voltage behavior is different from the other parameter’s response. The open circuit voltage monotonously decreases as the thickness of NPB increases. Nevertheless, the short-circuit current, fill-factor, and power-conversion efficiency demonstrate a very similar response, where all these photovoltaic parameters and most importantly the power-conversion efficiency are slightly improved from 10 to 100 nm approximately and continuously degraded when the thickness of NPB increases from 100 to 1000 nm. Therefore, from the above results, it can be justified that the 100 nm is the most optimum thickness of NPB (gives maximum power-conversion efficiency ~16.5%) as the hole-transport layer for the current proposed Ag/BCP/PCBM/Cs_2_TiBr_6_/NPB/ITO perovskite solar cell.

### 3.3. Optimization of Cs_2_TiBr_6_ as the Absorber Layer

**Figure 4:**
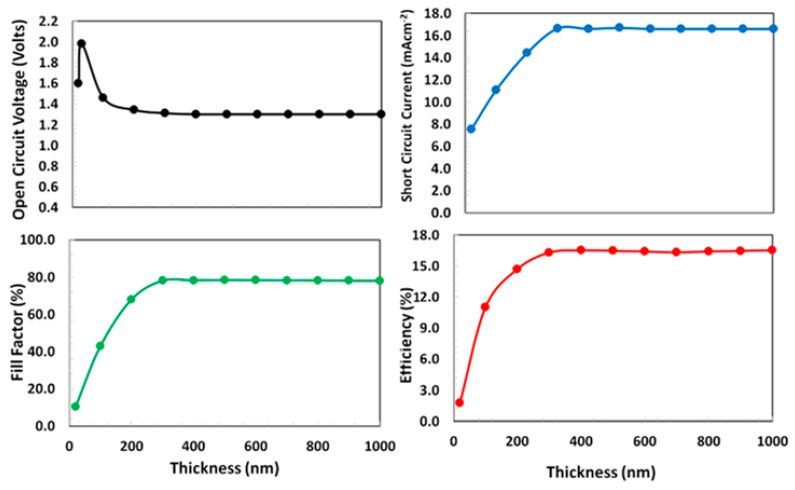
(**a**) Open-circuit voltage, (**b**) short-circuit current, (**c**) fill-factor, and (**d**) power-conversion efficiency of proposed solar cell Ag/BCP/PCBM/Cs_2_TiBr_6_/NPB/ITO as a function of Cs_2_TiBr_6_ thickness from 10 to 1000 nm.

The thickness optimization of the perovskite absorber layer is considered as one of the most crucial parts for the overall design of the proposed device for efficient performance. Either a very thin or very thick perovskite layer, is problematic in nature for the photovoltaic application. The thin perovskite layer offers poor photon absorption, while the thick perovskite layer compromises higher electron-holes recombination and hence degrades the photovoltaic response. Therefore, the optimum absorber layer thickness is the balance trade-off between these two factors. For the determination of design thickness of absorber layer, the optimized thickness of both PCBM (400 nm) and NPB (100 nm) layers and interface defect states were used in the proposed device and then a series of photo current-voltage simulations were carried-out. The photovoltaic parameters such as open-circuit voltage, short-circuit current, fill-factor, and power-conversion efficiency were calculated by varying the Cs_2_TiBr_6_ perovskite layer thickness from 10 to 1000 nm, as shown in Figure 4. From the figure, it is observed that photovoltaic parameters such as the fill-factor, short-circuit current, and power-conversion efficiency follow one trend, while the open-circuit voltage follows different trends from the others. The fill-factor, short-circuit current, and power-conversion efficiency sharply rise and then saturate at nearly 350 nm of the absorber layer thickness, as shown in Figure 4. However, the open-circuit voltage shows a little different behavior, it sharply increases at 50 nm and then gradually decreases and becomes stable at nearly 200 nm of the absorber layer thickness. As the proposed device reaches the maximum power-conversion efficiency at 350 nm of the absorber layer thickness, therefore, it can be inferred from the above discussion that the 350 nm can be estimated as the optimized thickness (gives maximum power-conversion efficiency ~16.5%) for the perovskite absorber layer for the proposed solar cell.

### 3.4. Photovoltaic Response of Proposed Solar Cell

**Figure 5:**
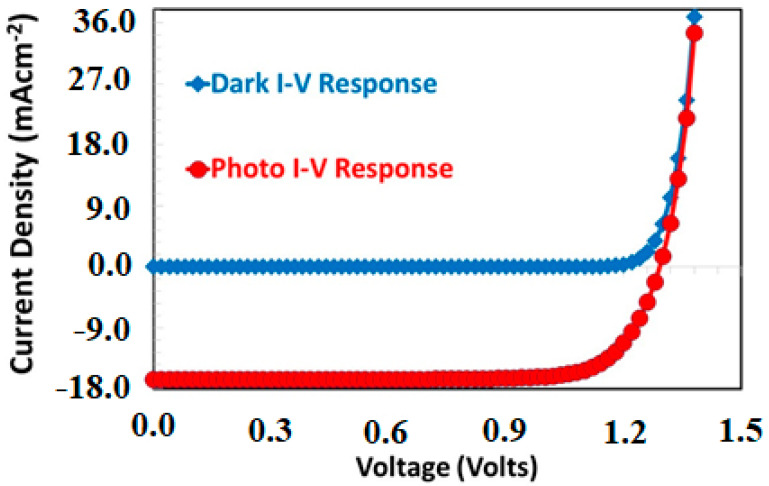
The simulated dark and AM 1.5 photovoltaic current responses of the proposed Ag/BCP/PCBM/Cs_2_TiBr_6_/NPB/ITO solar cell as a function of applied voltage.

Figure 5 shows the simulated current-voltage responses of the proposed solar cell both in the dark and in the presence of illumination, where the optimized thickness of PCBM (400 nm), NPB (100 nm), and Cs_2_TiBr_6_ (350 nm) as the electron-transport layer, hole-transport layer, and absorber layer were already incorporated in the simulation with interface defects states as discussed above. Both curves demonstrate the characteristics behavior of a photovoltaic diode response. In the dark current-voltage response, the proposed solar-cell shows a very low current passing-through the device especially at an earlier stage of the applied voltages. After the threshold voltage (approximately at 1.1 volts) both electrodes Ag and ITO started to inject relatively a large number of electrons and holes as the forward bias current [38,39]. Using the standard technique reported in the literature, the diode ideality factor was estimated as 1.3, which reflects the quality of the proposed diode as a solar cell [40,41].

Similarly, the proposed solar cell is also simulated for the photovoltaic response in the fourth quadrant operating between the open circuit and short circuit current, where a large amount of current flows when the solar-cell is illuminated, but this illuminated current is in the opposite direction compared to the dark current-voltage response, as shown in Figure 5. From the figure, it is clearly observed that the optimized device shows a reasonable photovoltaic response under the AM 1.5 illumination. As a result of this characterization, the photovoltaic parameters such as open-circuit voltage, short-circuit current. and fill-factor are extracted from the figure as 1.29 volts, 16.66 mA·cm^−2^, and 78.11%, respectively. All these photovoltaic parameters lead to the maximum power-conversion efficiency for the optimized proposed solar cell up to 16.85%. This efficiency is better than the previously reported power-conversion efficiency of a doped-free, lead-free perovskite solar cell [42].

### 3.5. External Quantum Efficiency of Proposed Solar Cell

**Figure 6:**
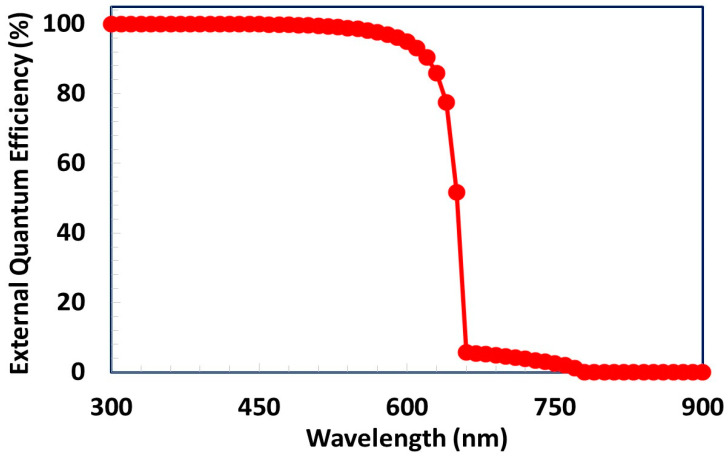
External quantum efficiency response as a function of incident photon wavelength for the proposed solar cell.

The external quantum efficiency response of the proposed Ag/BCP/PCBM/Cs_2_TiBr_6_/PTAA/ITO solar cell was simulated as a function of photons wavelength and the result is shown in Figure 6. The quantum efficiency for a photovoltaic device can be defined as the fraction of the free carriers collected from the respective electrodes to the total number of incident photons of a given wavelength (or energy) onto the top surface of the solar cell. Mathematically, the quantum efficiency and short-circuit current (*J_sc_*) are directly related to each other as a function of photon wavelength (*φ* (*λ*)) and can be expressed as [43,44]

(8) $$J_{SC}=q\;\int \varphi \left(\lambda \right)\;QE\left(\lambda \right)d\lambda$$

Here, we theoretically analyzed the external quantum efficiency of the proposed solar cell as a function of wavelength from 300 to 1000 nm, as shown in Figure 6. For simplicity, the observed external quantum efficiency response can be classified into two well-define regions. (i) Region I: Between 300 to 700 nm, (ii) Region II: Above 700 nm. In region I, the reduction of quantum efficiency is generally observed due to the reflection of photons as well as the low carrier diffusion length. As Cs_2_TiBr_6_ has a very high career diffusion length, therefore the excellent quantum efficiency response (>95%) is observed compared to the ideal quantum efficiency (100%) for region I [4]. It can be clearly demonstrated that the front-end (ITO) of proposed solar cell offers low optical reflections of photons. Hence, the proposed device shows the maximum external quantum efficiency for region I, which is a reasonably well-accepted value, as shown in Figure 6. In addition, as the energy band gap of Cs_2_TiBr_6_ is 1.55 eV, consequently, no photons are absorbed inside the proposed device for the higher photon’s wavelength for region II, as shown in Figure 6.

### 3.6. Thermal Stability of the Proposed Solar Cell

**Figure 7:**
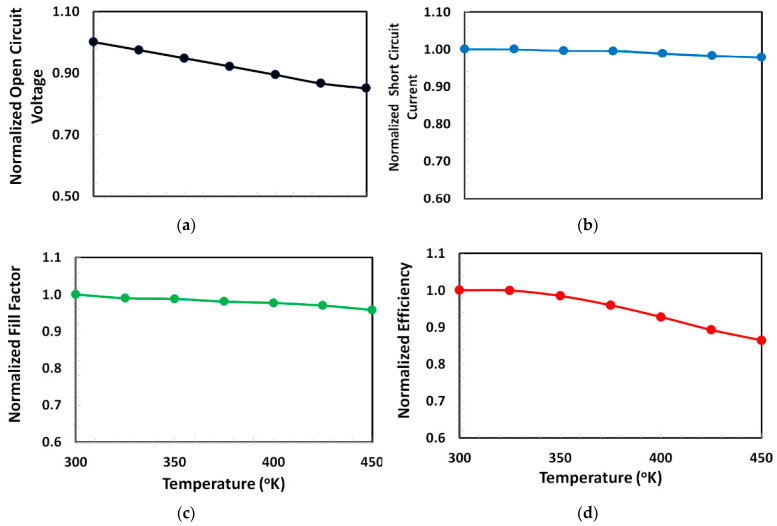
Degradation response of (**a**) normalized open-circuit voltage, (**b**) normalized short-circuit current, (**c**) normalized fill-factor, and (**d**) normalized power-conversion efficiency of the proposed solar cell Ag/BCP/PCBM/Cs_2_TiBr_6_/NPB/ITO as a function of ambient temperature varied from 300 to 450 °K.

The perovskite based solar cell has many types of defects, which in turn leads to some issues such as high carrier recombinational losses, degradation of interfacial contacts, and hence poor photovoltaic stability. The photovoltaic degradation becomes further aggravated when the perovskite solar cell is exposed to the higher ambient temperature. Therefore, the proposed solar cell is further characterized for the stability analysis by calculating the variation of photovoltaic parameters as a function of ambient temperature varied from 300 to 450 °K, as shown in Figure 7. From the figure, it is clearly observed that all the photovoltaic parameters are gradually degraded at higher temperature, but the rate of thermal degradation is different for each photovoltaic parameter. For a given temperature range if a linear model is applied for the relation between the normalized photovoltaic parameter and ambient temperature then their slope can be used as the rate of degradation (per °K) for a given photovoltaic parameter. From the figure, it is realized that both the short-circuit current and fill-factor shows very slow thermal degradation (rates of degradation are 2 × 10^−4^ and 3 × 10^−4^ per °K, respectively), while the open-circuit voltage and power-conversion efficiency also show very slow thermal degradation but higher than those in the short-circuit current and fill-factor for the proposed solar cell (rates of degradation are 1 × 10^−3^ and 1.5 × 10^−3^ per °K, respectively). Therefore, it can be stated from the above discussion that the proposed solar cell shows a relatively stable behavior within the given temperature range.

## 4. Conclusions

Toxicity and stability are the main hurdles for the commercialization of the perovskite solar cell. For this purpose, a planar p-i-n type perovskite solar cell is proposed, and the stability is improved by the proper selection of doping-free hole-transport (NPB) and electron-transport layer (PCBM). On the other hand, to avoid toxicity a novel lead-free perovskite compound cesium titanium (IV) bromide (Cs_2_TiBr_6_) is incorporated as the absorber layer and finally the Ag/BCP/PCBM/Cs_2_TiBr_6_/NPB/ITO solar cell was proposed as the lead and dopant free perovskite solar cell. For photovoltaic characterization, we theoretically analyzed, and optimized the proposed solar cell with the help of SCAPS 1D. After a comprehensive optimization of each layer, it is observed that the proposed perovskite solar cell can yield with maximum power-conversion efficiency up to 16.85%. We believe that the outcome of this study will help in the fabrication of lead and dopant free highly efficient and stable perovskite solar cell for future applications.

## Figures and Tables

**Table 1:** Materials parameters of BCP, PCBM, Cs_2_TiBr_6_, and NPB incorporated for these simulations are listed, where each layer thickness is just reported for the first estimation, which will further improve in later stages of the simulation.

Photovoltaic Parameters	Symbol	Unit	BCP	PCBM	Cs_2_TiBr_6_	NPB
Thickness	Th	nm	10	300	150	100
Energy Band Gap	Eg	eV	3.5	1.9	1.6	3
Electron Affinity	χ	eV	3.7	3.9	4.47	2.4
Dielectric Permittivity (Relative)	ε	-	10	4	10	3
Effective Density of States at Valence Band	N_V_	cm^−3^	2.2 × 10^18^	2.2 × 10^21^	1 × 10^19^	1 × 10^21^
Effective Density of States at Conduction Band	N_C_	cm^−3^	1.8 × 10^18^	1.8 × 10^20^	1 × 10^19^	1 × 10^21^
Hole Thermal Velocity	Ve	cm/s	1 × 10^7^	1 × 10^7^	1 × 10^7^	1 × 10^7^
Electron Thermal Velocity	Vh	cm/s	1 × 10^7^	1 × 10^7^	1 × 10^7^	1 × 10^7^
Electron Mobility	μe	cm^2^/V·s	2 × 10^−2^	1 × 10^−1^	44	6.1 × 10^−5^
Hole Mobility	μ_h_	cm^2^/V·s	2 × 10^−3^	1.5 × 10^−2^	2.5	6.1 × 10^−4^
Uniform Shallow Donor Doping	Nd	cm^−3^	1 × 10^21^	1 × 10^20^	1 × 10^13^	1 × 10^13^
Uniform Shallow Acceptor Doping	Na	cm^−3^	1 × 10^10^	1 × 10^13^	0	1 × 10^16^
Defect Density	N_t_	cm^−3^	1 × 10^14^	1 × 10^14^	1 × 10^17^	1 × 10^15^

**Table 2:** Interface traps parameters between the Cs_2_TiBr_6_ and NPB layer.

Parameters	Unit	Cs_2_TiBr_6_/NPB
Defect Type	-	Neutral
Capture cross section for electron	cm^−3^	1 × 10^14^
Capture cross section for electron	cm^−3^	1 × 10^14^
Enerfetic Distribution	-	Single
Energy level with respect to Ev	eV	6.0 × 10^−1^
Characteristic Energy	eV	~0.1
Defect Density	cm^−3^	4.5 × 10^18^
